# Recent advances in preventing recurrent stroke

**DOI:** 10.12688/f1000research.23199.1

**Published:** 2020-08-20

**Authors:** J David Spence

**Affiliations:** 1 Western University, and Stroke Prevention & Atherosclerosis Research Centre, Robarts Research Institute, Ontario, Canada

**Keywords:** Stroke

## Abstract

Since a 2017 update, there have been important advances in stroke prevention. These include new evidence about nutrition, antiplatelet therapy, anticoagulation, lipid-lowering therapy, hypertension control, pioglitazone, and carotid endarterectomy and stenting. Evidence regarding toxic metabolites produced by the intestinal microbiome from egg yolk and red meat has important dietary implications, particularly for patients with impaired renal function, including the elderly. They should avoid egg yolk and red meat and limit the intake of animal flesh. Higher doses of folic acid may be needed for patients with the T allele of MTHFR, so it may not be sufficient to give vitamin B12 (methylcobalamin) alone, even in countries with folate fortification. There is now good evidence that lipid-lowering therapy is even more beneficial in the elderly than in younger patients; we should be using lipid-lowering therapy more intensively, often/usually combining statins with ezetimibe. There is new evidence that lower systolic blood pressure targets are better for most patients, but a subgroup with stiff arteries, a wide pulse pressure, and a diastolic pressure of <60 would be more likely to be harmed than helped by aiming for a systolic target of <120 mmHg. There is a better understanding of how the pharmacological properties of direct-acting oral anticoagulants and the metabolism of antiplatelet agents should inform decisions about the use of these agents. Pioglitazone markedly reduces the risk of stroke, both in diabetics and prediabetics; it should be used more widely. It is now clear that carotid endarterectomy is safer than stenting and that the difference is strongly affected by age. Most patients, and in particular older patients, would be better served by endarterectomy than stenting.

## Introduction

In a 2017 update ^1^, I discussed advances in hypertension, nutrition, anticoagulation, antiplatelet therapy, intracranial stenosis, percutaneous closure of patent foramen ovale, and lipid-lowering therapy. Readers are invited to study that review. In this update, I discuss more recent developments in nutrition, hypertension, antiplatelet therapy, anticoagulation, lipid-lowering therapy, carotid endarterectomy (CEA) and carotid artery stenting (CAS). Exercise, smoking cessation, and adherence are also important, but there have been few recent advances in those topics. A report from the Insulin Resistance Intervention in Stroke (IRIS) trial reported a 34% reduction of stroke within 5 years among participants who quit smoking ^2^. A Swedish study reported important improvements in risk scores, presumed to be a result of improved adherence, among patients with one or more risk factors attending a prevention program who were shown images of their carotid plaque ^3^.

## Nutrition

In 2017, I discussed the important advantages of the Mediterranean diet for stroke prevention. Since then, there have been developments regarding the consumption of cholesterol, meat, and eggs and the dietary implications of the interaction between renal function and the intestinal microbiome.

### Meat, cholesterol, and egg consumption

Dietary cholesterol increases cardiovascular risk ^4^, so it seems obvious that, as egg yolk is very high in cholesterol content, the consumption of eggs would increase cardiovascular risk. Similarly, the consumption of meat, which is high in cholesterol and saturated fat, should also increase cardiovascular risk. This has been difficult to demonstrate, and indeed some observational studies suggest that egg and meat consumption are not harmful. Such findings are very likely based on indication bias and both measured and unmeasured confounders ^5^ and are not biologically plausible. The reason it has been hard to demonstrate harm from eggs and meat is that the US diet is so bad that the AHA reported in 2015 that only 0.1% of Americans consume a healthy diet ^6^. On that background, it is hard to show that *anything* makes it worse. On the other hand, in Greece, where the Mediterranean diet is the norm, the harm from egg consumption was more obvious. Among Greek diabetics, an egg a day increased coronary risk fivefold, and even 10 grams/day of egg (a sixth of a large egg) increased coronary risk by 54% ^7^.

Furthermore, besides cholesterol, egg yolk and red meat also contain high levels of dietary precursors of trimethylamine N-oxide (TMAO). High levels of TMAO strongly predicted the 3-year risk of stroke/myocardial infarction/vascular death among >4,000 patients referred to the Cleveland Clinic for coronary angiography ^8^. We reported in 2018 that plasma levels of TMAO and three other toxic metabolites produced by the intestinal microbiome were significantly higher in patients with severe atherosclerosis not predicted by risk factors (“unexplained atherosclerosis”) than in patients with a protected phenotype (little or no carotid plaque despite high levels of coronary risk factors) ^9^. Hazen’s group recently reported that phenylacetylglutamine increases cardiovascular risk via effects on adrenergic receptors ^10^.

I recently reviewed evidence that vegetarian diets reduce cardiovascular risk. Perhaps the best evidence about this issue is presented by Zhong *et al*. ^11^, who reported that, in the US, on the basis of data from 29,615 participants during a median follow-up of 17.5 years, both cholesterol consumption and egg consumption increased cardiovascular risk in a dose-dependent manner.

### Interaction of renal function and toxic metabolites of the intestinal microbiome

Plasma levels not only of TMAO but also all of the toxic metabolites of the intestinal microbiome that we measured (TMAO, p-cresyl sulfate, hippuric acid, p-cresyl glucuronide, phenylacetylglutamine, and phenylsulfate), were significantly higher among study participants with an estimated glomerular filtration rate (eGFR) of <66 mL/minute/1.73 m ^2^ ^12^. Such levels of eGFR are the norm for patients aged over 75 ^13^. This means that patients with impaired renal function, including the elderly, should avoid egg yolk and red meat and limit their intake of animal flesh.

### B vitamins for lowering of homocysteine

In the previous review ^1^, I summarized evidence showing that B vitamins to lower homocysteine do indeed reduce the risk of stroke. In the early studies, harm from cyanocobalamin among participants with renal failure obscured the benefit. For that reason, we should use methylcobalamin instead of cyanocobalamin ^14^. I had been under the impression that folate fortification of the grain supply in North America meant that we needed to give only B12 supplements to reduce the risk of stroke. However, recent studies indicate that patients with the T allele of methylene tetrahydrofolate reductase (MTHFR) probably need higher doses of folate than would be obtained from fortification of the grain supply ^15^. This means that we will need to reconsider higher doses of folic acid in addition to B12 supplements and perhaps vitamin B6 and riboflavin ^16, 17^ as well. Further study is needed; a trial of methylcobalamin plus folic acid (and perhaps pyridoxine) versus placebo would be important though unlikely to be funded. Subgroups of patients most likely to benefit would be those with metabolic B12 deficiency, which is very common and usually missed, and patients with the T allele of MTHFR.

## Antiplatelet agents

There are a couple of important developments in antiplatelet therapy. Grosser *et al*. ^18^ reported that apparent “aspirin resistance” was due to enteric coating. We should probably be using chewable or uncoated aspirin, not coated aspirin.

The other major issue is that clopidogrel, a prodrug, requires activation by CYP2C19. Patients with a loss-of-function variant of CYP2C19 have reduced benefit of clopidogrel ^19^. That loss-of-function variant is present in ~30% of European and >50% of Chinese patients. We probably should stop using clopidogrel and switch to prasugrel or ticlopidine. A recent study indicated that prasugrel was superior to ticlopidine ^20^.

There have been a number of other advances in antiplatelet therapy, including combination of aspirin with rivaroxaban, as well as dual antiplatelet therapy for the prevention of recurrent stroke, which are not discussed here owing to word count limits.

## Anticoagulants

### Properties of direct-acting oral anticoagulants

Although there are not randomized controlled trial (RCT) head-to-head comparisons across the class of direct-acting oral anticoagulants (DOACs), there are important differences in their pharmacokinetic properties that should be considered in choosing which one to use. These are summarized in Table 1 ^21^.

**Table 1.** Characteristics of direct-acting oral anticoagulants (DOACs).

Characteristic	Rivaroxaban	Dabigatran	Apixaban	Edoxaban
Target	Factor Xa	Factor IIa	Factor Xa	Factor Xa
Prodrug	No	Yes	No	No
Dosing	Once daily	Twice daily	Twice Daily	Once Daily
Bioavailability	80%–100%	6.5%	50%	62%
Half-life	5–13 hours	12–14 hours	8–15 hours	10–14 hours
Renal clearance	~33%	85%	~27%	~50%
Cmax	2–4 hours	1–2 hours	3–4 hours	1–2 hours
Interactions	Strong inhibitors of CYP3A4 and P-gp	P-gp inhibitors	Strong inhibitors of CYP3A4 and P-gp	P-gp inhibitors

CYP3A4, intestinal cytochrome P450 3A4; P-gp, P-glycoprotein.

This table was reproduced from Cardioembolic stroke: everything has changed, Spence JD, 3:76-83, 2018 ^21^ with permission from BMJ Publishing Group Ltd.

Dabigatran is the most renally excreted of the DOACs, so it is unsuitable for patients with impaired renal function. Since the mean eGFR in patients older than 80 is <60 mL/minute/1.73 m ^2^ ^13^, this means that dabigatran is probably not a good choice for elderly patients. Dabigatran also has by far the lowest bioavailability, which makes it subject to large changes in blood levels with small changes in absorption or with drug interactions. It has a narrow therapeutic range, so it has been suggested that blood levels of dabigatran should probably be monitored ^22^, thus negating one of the major advantages over warfarin. Rivaroxaban is not longer acting than the other DOACs, so it probably should not be administered once daily. Tellingly, recent studies with rivaroxaban have used twice-daily dosing ^23^. As edoxaban also does not have a half-life longer than that of other DOACs, it too should probably not be taken once daily.

**Figure 1.**
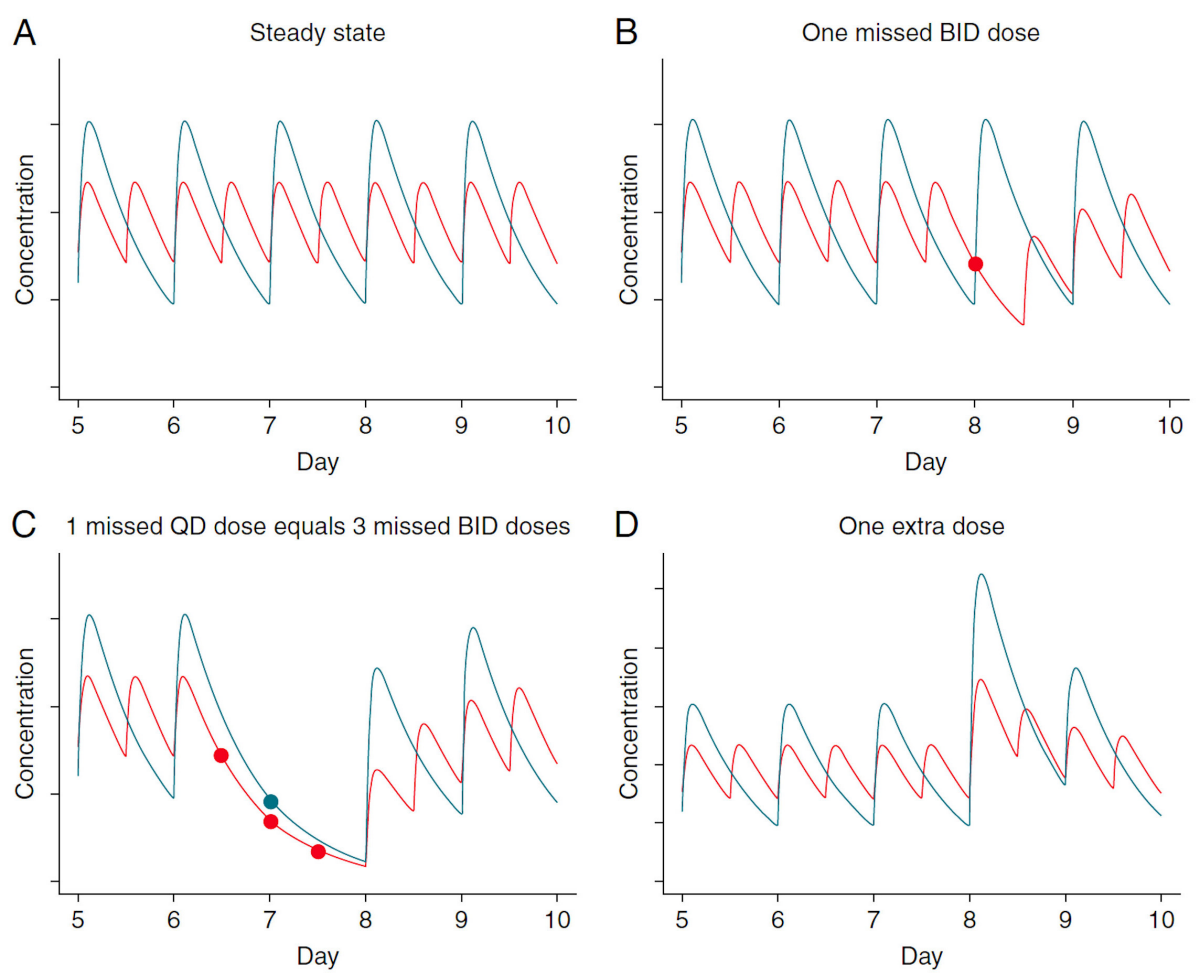
Once-daily vs. twice-daily dosing: difference between intake and predicted biological impact in general. Different patterns of non-adherence lead to different exposition to “risk” between once-daily and twice-daily drugs. These graphs illustrate the theoretical pharmacokinetic proﬁles of a dose X administered once-daily (QD) and a dose X/2 administered twice-daily (BID) for a drug with a half-life of about 12 hours and a Tmax of 3 hours. ( **A**) The peak-to-trough ratio is much smaller for the BID than the QD dosing. ( **B**) The concentration after a single missed BID dose (red dot) is similar to the expected trough concentration of QD dosing, suggesting that missing a single dose of a twice-daily dosing regimen should not be therapeutically critical. ( **C**) The pharmacological equivalent of missing a single dose in a once-daily regimen (blue dot) is missing three consecutive doses (red dots) of a twice-daily dosing regimen. ( **D**) Taking an extra dose results in a much higher peak for the QD than for the BID dosing regimen. This figure was reproduced from Heidbuchel H, Vrijens B. Non-vitamin k antagonist oral anticoagulants: Considerations on once- vs. Twice-daily regimens and their potential impact on medication adherence. EP Europace. 2015;17:1317-1318 ^24^ by permission of Oxford University Press.

Vriejens and Heidbuchel reviewed the advantages of twice-daily dosing with apixaban vs. once-daily dosing with rivaroxaban. With twice-daily dosing, the blood levels of the drug stay within the therapeutic range throughout the day, instead of swinging from too high to too low, and there is less impact of a missed dose or an extra dose. One missed dose of rivaroxaban would be equivalent to missing three doses of apixaban, and an extra dose of rivaroxaban has a much bigger effect, with risk of bleeding, than an extra dose of apixaban ^24^ ( Figure 1).

This may account for recent reports that apixaban is less likely to cause GI bleeding than rivaroxaban ^25^, more efficacious with regard to ischemic stroke or systemic embolism, and safer with regard to bleeding than rivaroxaban ^26^. In a study comparing risk of hip fracture among patients taking warfarin vs. DOACs, apixaban had the lowest risk of hip fracture ^27^.

### Misclassification of large artery atherosclerosis in embolic stroke of unknown source

Antiplatelet agents are not anticoagulants; they prevent the aggregation of platelets in fast-flowing blood in arteries (white thrombus) but do not prevent “red thrombus”, which forms in the setting of stasis, with fibrin polymerization and entrapped red blood cells ^28, 29^. For that reason, anticoagulants are more effective for preventing cardioembolic stroke.

In the past, when vitamin K antagonists such as warfarin were the only drugs available for anticoagulation, it was understandable that the paradigm would essentially be that one would never prescribe an anticoagulant without proving the presence of a cardioembolic source such as atrial fibrillation. However, since DOACs are not significantly more likely than aspirin to cause severe bleeding ^30, 31^, the paradigm should change. There are good reasons to think that in patients in whom a cardioembolic source is strongly suspected, it would be more prudent to prescribe a DOAC than an antiplatelet agent ^32^.

Several trials to test that hypothesis failed to show a benefit of anticoagulation in patients with embolic stroke of unknown source (ESUS); however, it is very likely that misclassification of large artery disease may have accounted for that. When large artery atherosclerosis (LAA) is defined as a 50% carotid stenosis, it will miss 79% of patients classified as LAA by a high carotid plaque burden ^33^. Since antiplatelet therapy is more efficacious in LAA, failing to exclude such patients may be why anticoagulation was not superior in ESUS. Importantly, a meta-analysis that was included in a substudy of one of those trials showed that anticoagulation was superior to antiplatelet therapy for patients with patent foramen ovale ^34^.

## Hypertension

### Physiologically individualized therapy based on renin/aldosterone phenotyping

Reasons why physiologically individualized therapy based on renin/aldosterone phenotyping significantly improves blood pressure control ^35^, discussed in the 2017 review, have become more apparent. The physiology of salt and water retention was reviewed in 2018 ^36^. A study of hypertension in foreign-born vs. US-born blacks revealed that African-Americans have nearly twice the rate of hypertension ^37^, supporting the African Diaspora theory proposed by Grim ^38^ and others: the very high mortality rate during the Atlantic crossing in slave ships, from diarrhea, vomiting, and sweating in the heat below decks, created a selective advantage for genetic causes of salt and water retention. It is now apparent that at least six genetic variants predispose to a primary aldosteronism/inappropriate aldosterone secretion phenotype (low renin/low aldosterone), best treated with an aldosterone antagonist such as spironolactone or eplerenone, and at least six predispose to a Liddle phenotype (low renin/low aldosterone), best treated with amiloride ^39^. An important cause of resistant hypertension is “Diagnostic Inertia” – failure to ask the question “What is the physiological driver of the hypertension in this patient?”. Plasma renin and aldosterone should be measured in a stimulated condition (i.e. while taking a diuretic, angiotensin receptor antagonist, or angiotensin-converting enzyme inhibitor), and the results should be interpreted in light of the class(es) of medication being taken at the time of blood sampling ^40^.

### Lower blood pressure targets in frail elderly patients

In the wake of the Standard Trial of Intensive versus Blood-Pressure Control (SPRINT) trial, among participants with increased cardiovascular risk but without diabetes ^41^, lower blood pressure targets (a systolic pressure of <120 mmHg) are being recommended for blood pressure control. This approach may be well suited to most hypertensive patients, but there is an important exception. Patients with stiff arteries are at risk from systolic targets that are too low. The reasons include 1. stiff arteries widen pulse pressure, 2. stiff arteries increase the likelihood that the blood pressure measured by a cuff is actually much higher than the true (intra-arterial) pressure ^42^, and 3. most of coronary perfusion ^43^, and more than half of cerebral perfusion ^44^, occurs during diastole. For that reason, patients with a diastolic pressure of <60 mmHg with a pulse pressure of >60 mmHg have a doubling of subclinical myocardial ischemia ^43^ and a 5.85-fold increase in the risk of stroke ^45^. Frail elderly patients with stiff arteries (a pulse pressure of >60 mmHg) should not be treated to a target systolic pressure of <120 mmHg. A 2019 meta-analysis of standard vs. intensive blood pressure control for secondary stroke prevention supported a blood pressure target of less than 130/80 mmHg ^46^.

## Lipid-lowering therapy

A key part of stroke prevention is lipid-lowering therapy; this is particularly important in patients with LAA. Amarenco *et al*. recently reported that lower target LDL-C significantly reduced the risk of recurrent stroke ^47^. Participants randomized to a lower target LDL-C of <70 mg/dL (1.8 mmol/L) vs. a target range of 90 mg to 110 mg/dL (2.3 to 2.8 mmol/L) had a significantly lower risk of the primary endpoint (ischemic stroke, myocardial infarction, new symptoms leading to urgent coronary or carotid revascularization, or death from cardiovascular causes; adjusted hazard ratio [HR] 0.78; 95% confidence interval [CI] 0.61–0.98; *P* = 0.04). A frequently missed opportunity is in adding ezetimibe to statins; ezetimibe more than doubles the effect of statins, with fewer adverse effects than the use of high-dose statins ^48^. The recent European guidelines recommend the addition of ezetimibe in high-risk patients who do not achieve a target LDL-C of 140 mmol/L (53 mg/dL) ^49^. In the guideline, high risk was defined as having a previous cardiovascular event, but patients with asymptomatic carotid stenosis ^50^, a high coronary calcium score ^51^, or a high carotid plaque burden ^52^ are also at very high risk ^52^. A number of new lipid-lowering therapies are in development ^53^.

Two other recent developments are from the 2019 European guideline on the management of dyslipidemia, with consensus guidelines recommending a lower target LDL-C of 1.4 mmol/L (53 mg/dL) and the use of ezetimibe in patients who do not achieve target levels with statin alone. Using ezetimibe with statins is an important measure. Because statins and ezetimibe work by different mechanisms, the combination is synergistic. Ezetimibe more than doubles the lowering of LDL-C by statins. In patients who do not tolerate full doses of statins, adding ezetimibe will achieve the effect of high-dose statins with fewer adverse effects (probably the only truly causal adverse effect of ezetimibe is loose bowel movements, rarely diarrhea).

The importance of keeping patients on their lipid-lowering therapy was shown in the 2020 trial by Amarenco *et al*. ^47^. Patients with ischemic stroke and atherosclerosis were randomized to a lower target LDL-C of less than 1.8 mmol/L (70 mg/dL) vs. a target of 2.3 to 2.8 mmol/L (90 mg to 110 mg/dL). At sites where patients were in the target range for >50% of the time, major cardiovascular events were reduced by 36% (HR 0.64, 95% CI 0.48–0.85); at sites where participants were at target <50% of the time, there was no reduction in major cardiovascular events (HR 1.14, 95% CI 0.75–1.73). There is a major problem with patients stopping statins for the wrong reasons (often influenced by internet nonsense). A review in 2016 ^48^ discussed approaches to helping patients stay on statins; adding ezetimibe is a cornerstone of that effort.

An important advance is that there is now good evidence that lipid-lowering therapy is even more beneficial in the elderly than in younger patients. I recently reviewed this issue ^54^. In the past, there was a tendency to withhold statins in older patients on the basis that since patients over the age of 80 years were not enrolled in randomized trials, there was no direct evidence of benefit; but, then again, there was also no evidence of harm! As discussed by Mortensen and Falk ^55^, because the elderly are at higher risk of cardiovascular events, the benefit of lipid lowering should be even greater in old patients than in younger patients, with a greater absolute risk reduction and a smaller number needed to treat (NNT). This has indeed been shown to be the case in an RCT of adding ezetimibe to simvastatin: the NNT in patients aged over 75 was only 11 vs. 125 in patients younger than 75. In an RCT in Japan, patients with a mean age of 80.6 years at baseline were randomized to diet alone or diet plus ezetimibe. Over a median follow-up of 4.1 years, there was a 34% reduction of composite cardiovascular events and a 40% reduction of combined coronary events ^56^.

A scientific statement from the American Heart Association in 2019 reviewed the evidence that statins do not cause most of the adverse effects that have been attributed to them ^57^. Collin *et al*. point out that most such putative adverse effects based on observational studies are the result of indication bias or confounders, and they are refuted by meta-analysis of randomized trials of statins in very large numbers of patients ^58^. The GREACE trial established that patients with elevated liver enzymes actually do better if they continue statins; “transaminitis” is probably due to fatty liver ^59^. The European Guideline ^49^ now recommends against monitoring of liver function in patients on statins and against stopping statins on account of “transaminitis”. I recently reviewed the issue of intracerebral hemorrhage (ICH). The widespread belief that statins cause ICH was an artefact of an inappropriate intention-to-treat analysis of the Stroke Prevention by Aggressive Reduction in Cholesterol Levels (SPARCL) RCT, in which ~25% of patients randomized to placebo crossed over to statin, and many patients randomized to statin stopped taking it. The proof that atorvastatin could not have caused ICH is that there was a greater reduction of ischemic stroke, but no increase in ICH, among SPARCL participants who achieved a 50% reduction of LDL-C (i.e. they were taking the medication) ^60^. Even if there were a slight increase in ICH with statins, there would be far greater harm from withholding or stopping statins than continuing them ^61^.

Lipid-lowering therapy should be intensive, should frequently (or usually) be combined with ezetimibe, and should not be withheld on account of age or misplaced fear of ICH. In some patients, if the very high cost is not an issue, therapies directed at proprotein convertase subtilisin/kexin type 9 (PCSK9) might be considered.

## Pioglitazone

Diabetes and insulin resistance are risk factors for stroke. Pioglitazone, a weak agonist of peroxisome proliferator-activated receptor-α (PPAR-α) and a potent agonist of PPAR-γ, reduces insulin resistance and has anti-atherosclerotic effects ^62^. In 2016, the IRIS trial reported a 23% reduction in recurrent stroke with pioglitazone over 5 years in the intention-to-treat analysis, but adherence was suboptimal in many patients, mainly because of fluid retention and weight gain.

**Figure 2.**
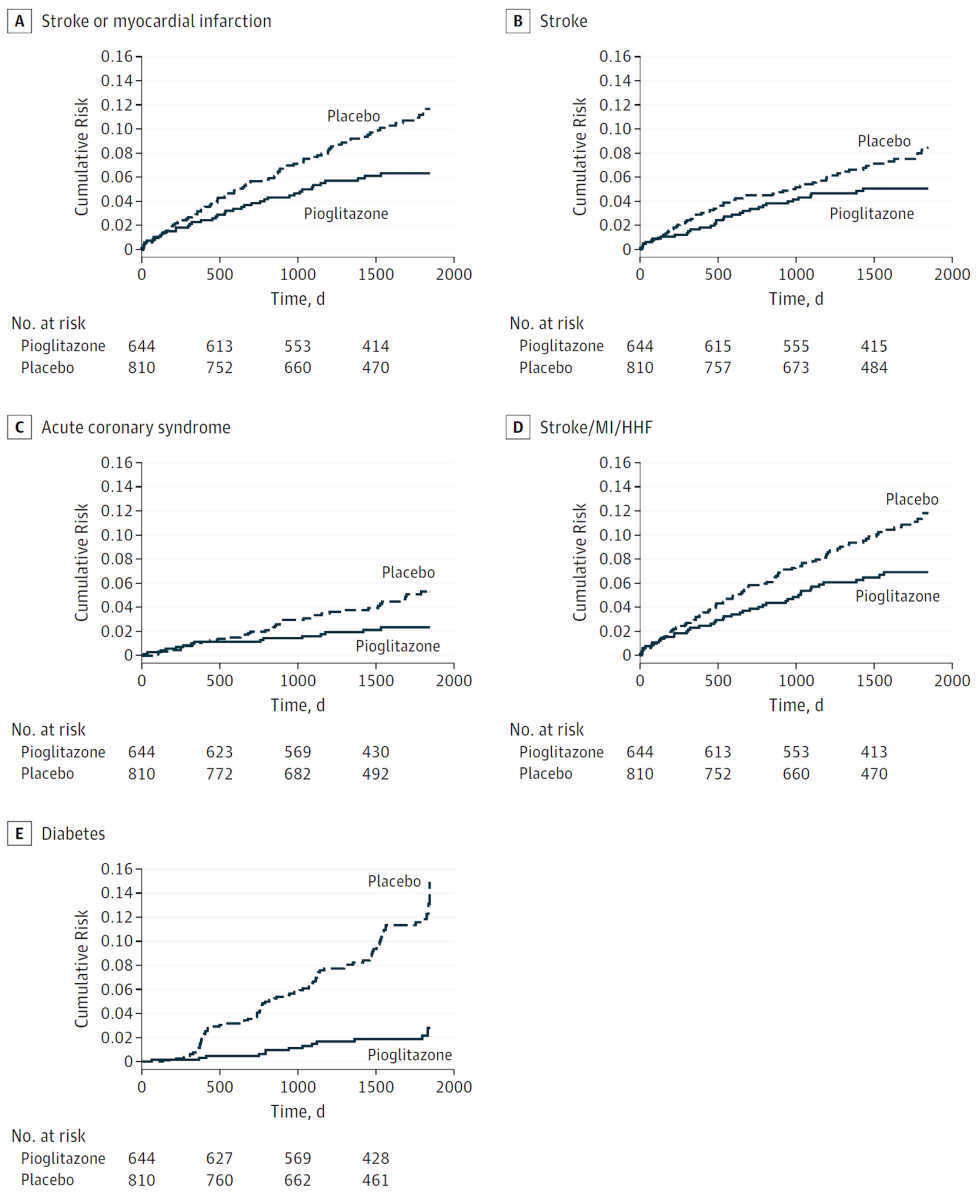
Effect of pioglitazone in patients with stroke and prediabetes: results in patients who took >80% of the protocol dose. ( **A**) Stroke or myocardial infarction (hazard ratio [HR] 0.57; 95% confidence interval [CI] 0.39–0.84; *P* = 0.004). ( **B**) Stroke (HR 0.64; 95% CI 0.42–0.99; *P* = 0.04). ( **C**) Acute coronary syndrome (HR 0.47; 95% CI 0.26–0.85; *P* = 0.01). ( **D**) Stroke/myocardial infarction (MI)/hospitalization for heart failure (HHF) (HR 0.61; 95% CI 0.42–0.88; *P* = 0.008). ( **E**) New-onset diabetes (HR 0.18; 95% CI 0.10–0.33; *P* <0.001). This figure was reproduced from Spence JD, Viscoli CM, Inzucchi SE, Dearborn-Tomazos J, Ford GA, Gorman M, *et al*. Pioglitazone therapy in patients with stroke and prediabetes: A post hoc analysis of the iris randomized clinical trial. JAMA Neurol. 2019;76:526-535 ^62^ with permission from the American Medical Association.

A meta-analysis in 2017 reported a 42% reduction of recurrent stroke with pioglitazone among patients with stroke and diabetes, insulin resistance, or prediabetes ^63^. In 2019, a substudy of the IRIS trial was conducted among participants with insulin resistance defined by a glycosylated hemoglobin (A1C) level of 5.7 to 6.4% or fasting plasma glucose level of 5.55 to 6.94 mmol/L (100mg/dL to 125mg/dL). Among those with good adherence (taking >80% of the protocol dose), stroke or myocardial infarction was reduced by 43% in 5 years, recurrent stroke was reduced by 36%, and new-onset diabetes was reduced by 82% ^62^ ( Figure 2).

Pantoni, in an accompanying editorial ^64^, noted that pioglitazone is probably underutilized, in part because of a probably unjustified belief that it increases the risk of bladder cancer. There is also a risk of fractures with pioglitazone, but the NNT to prevent one stroke or myocardial infarction with pioglitazone is only 24, the NNT to prevent one case of new-onset diabetes is only 12, and the number needed to harm (NNH) to cause one serious fracture is 125 ^62^. Pioglitazone should be used more widely for stroke prevention, both in diabetes and in prediabetes/metabolic syndrome.

## Carotid endarterectomy and stenting

**Figure 3.**
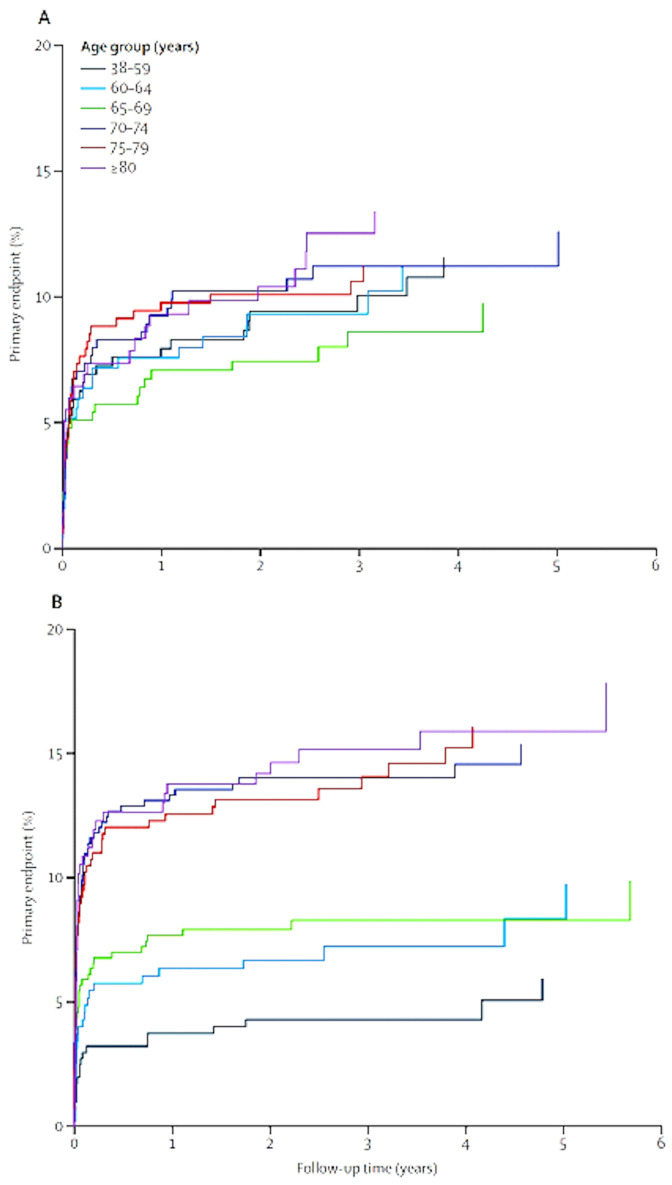
Estimated event rates for patients assigned to CEA ( **A**) and CAS ( **B**) by age stratum. The age groups are shown by the color key. Events plotted during the ﬁrst 120-day periprocedural period include stroke in either hemisphere plus deaths, whereas events during the postprocedural period include ipsilateral stroke only. Patients were censored at 6 years for the graphical presentation of event rates. CAS, carotid artery stenting; CEA, carotid endarterectomy. This figure was reprinted from The Lancet, 387, Howard G, Roubin GS, Jansen O, Hendrikse J, Halliday A, Fraedrich G, *et al*., Association between age and risk of stroke or death from carotid endarterectomy and carotid stenting: A meta-analysis of pooled patient data from four randomised trials, 1305-1311., 2016 ^67^ with permission from Elsevier.

Recent advances include two important meta-analyses. Brott *et al*. ^65^ reported from a pooled analysis of individual data from 4,754 participants in symptomatic carotid stenosis that CEA was superior to CAS; the risk of stroke or death or subsequent ipsilateral stroke with CEA was 2.8% (95% CI 1.1–4.4) vs. 4.1% (2.0–6.3) with CAS at follow-up times up to 10 years. These outcomes were dominated by periprocedural events ^66^. It is now clear from a meta-analysis by Howard *et al*. ^67^ that CEA is preferable for most older patients ( Figure 3). “CAS should be reserved for selected patients. Factors that would favour CAS could include younger age, specific anatomical features (such as a stenosis that is in the very distal internal carotid artery), lack of tortuosity of the arteries leading to the stenosis, absence of or only minimal plaque calcification, presence of local tissue scarring due to previous surgery or radiation, and conditions conferring a high medical risk for surgery (such as congestive heart failure, myocardial ischaemia, or severe pulmonary disease)” ^66^.

## Summary/conclusion

What does the future hold? Probably advances in transcarotid stenting will show the benefit of this approach over transfemoral or transradial stenting. New therapies for cholesterol-lowering will probably also improve stroke prevention, as well as improvements in antiplatelet therapy. What questions remain? Ongoing RCTs of stenting vs. endarterectomy vs. intensive medical therapy will likely provide answers about asymptomatic stenosis that may be applicable to symptomatic carotid stenosis; also, we need further evidence about anticoagulation in ESUS when the misclassification of LAA has been addressed. There have been many recent advances in stroke prevention; no doubt more are to come.
